# Ossification post-traumatique du ligament collatéral médial du genou: à propos d’un cas

**DOI:** 10.11604/pamj.2016.24.254.9723

**Published:** 2016-07-19

**Authors:** Hafid Arabi, Hicham Sellahi

**Affiliations:** 1 Equipe de Recherche Clinique et Epidémiologique de la Pathologie Ostéo-articulaire, UCH Mohammed VI, Faculty of Medicine and Pharmacy Marrakech, Cadi AAyad University, Maroc; 2 Service de Traumato-orthopédie, Hôpital Militaire Avicenne Marrakech, CHU Mohamed VI, Faculté de Médecine et de Pharmacie Marrakech, Université Kadi Ayad, Maroc

**Keywords:** Ossification hétérotopique, ligament collatéral médial, Heterotopic ossification, medial collateral ligament, Morocco (à enlever)

## Abstract

L’ossification hétérotopique est une ossification pathologique des parties molles. C’est une affection bénigne, récidivante et de survenue imprévisible. On décrit le cas d’un patient, traité pour une fracture du plateau tibial gauche avec mise en place d’une plaque vissée. A deux mois de l’opération, le patient a présenté une raideur du genou gauche. Le bilan radiologique standard a révélé une ossification du ligament latéral interne (LLI), confirmée par la tomodensitométrie. On ne sait pas actuellement les facteurs déterminant l’ossification des ligaments et tendons. Bien que les cliniciens prescrivent souvent des médicaments prophylactiques tels que les anti-inflammatoires et des séances de radiothérapie pour prévenir l’ossification hétérotopique des parties molles; la physiopathologie de l’ossification hétérotopique est peu connue.

## Abstract (en)

Heterotopic ossification is a pathological ossification of soft tissues. It is a benign, recurring disease with an unpredictable onset. We here report the case of a patient treated for a fracture of the left tibial tray with installation of a screwed plate. Two months after surgery the patient had a stiff left knee. Standard radiological examination showed ossification of the medial collateral ligament (MCL) confirmed by tomodensitometry. We do not currently know which factors determine the presence of tendon and ligamentous ossification. Although clinicians often prescribe prophylactic drugs, such as anti-inflammatory drugs and radiotherapy sessions to prevent heterotopic ossification in soft tissues, the physiopathology of heterotopic ossification is little known.

## Introduction

L’ossification hétérotopique est une affection bénigne touchant les éléments péri-articulaires ou parties molles. A travers ce cas, nous évoquons les différents mécanismes physiopathologiques. Une rééducation précoce, adéquate et continue après tout acte médico-chirurgical éviterait l’enraidissement articulaire.

## Patient et observation

**Figure 1:**
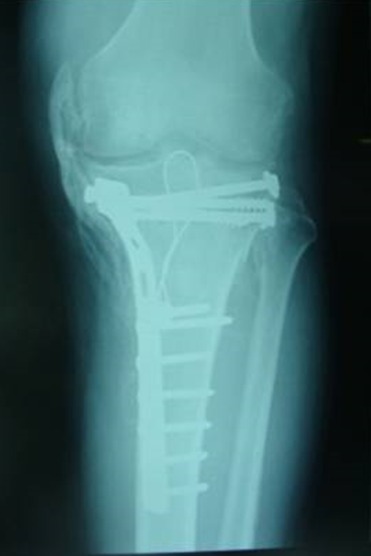
Pont osseux le long du compartiment interne longeant le LLI

**Figure 2:**
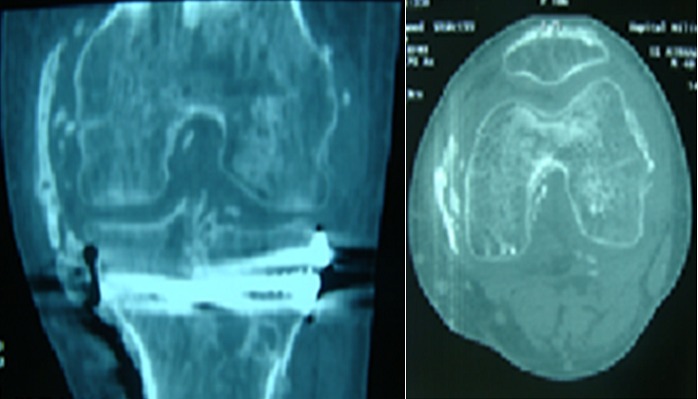
Tomodensitométrie: ossification du LLI

**Figure 3:**
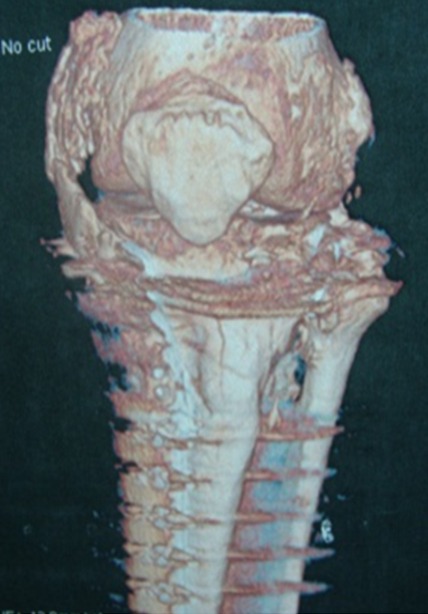
Tomodensitométrie en trois dimensions: ossification du LLI

Il s’agit d’un patient âgé de 37 ans, sans antécédent pathologie, roulant à une vitesse de 80 km/heure, il a eu un traumatisme du genou gauche après être entré en collision avec une voiture. Il a présenté une fracture fermée du plateau tibial gauche. Il n’y avait pas de traumatisme crânien. Il a été opéré à J 7 avec un abord antéro-médial et avec mise en place d’un matériel d’ostéosynthèse. Il était perdu de vue pendant 2 mois, au cours des quels, il a développé une raideur du genou. Ceci a motivé une consultation; l’examen a trouvé une cicatrice opératoire non inflammatoire et non adhérente, disparition des méplats du compartiment interne du genou gauche, on notait l’absence d’épanchement articulaire; l’amyotrophie du quadriceps était manifeste. Les amplitudes articulaires étaient: 0/10°/20°, avec un jeu articulaire de 10° et perte de la mobilité de la rotule. La radiographie standard a révélé un pont osseux le long du compartiment interne longeant le LLI (Figure 1); la phosphatase alcaline était normale. Pour complément de bilan, la tomodensitométrie a confirmé l’ossification du LLI (Figure 2 et Figure 3).

## Discussion

L’ossification des tendons et ligaments est une situation rare. L’ossification hétérotopique péri-articulaire ou des parties molles (OH) est le terme souvent utilisé dans la littérature pour décrire cette localisation d’ossification pathologique. Parler de l’ossification d’un constituant des parties molles (muscle, ligament, tendon….) est évoqué que lorsque la localisation est identifiée par les investigations radiologiques approfondies. La distinction nosologique entre l’ossification de ces constituants n’est pas claire. L’ossification hétérotopique a été décrite dans la chirurgie prothétique de genou [1, 2], du ligament croisé antérieur [3], ligament latéral du genou en post-traumatique [4] et du genou dans l’enclouage centromédullaire rétrograde (ECM) [5].

L’étiologie de l’ossification hétérotopique des parties molles est inconnue. Deux mécanismes étiopathogéniques sont évoqués [6, 7]. Dans le premier cas, il s’agit d’une greffe de cellules ostéoblastiques. La deuxième hypothèse fait intervenir la métaplasie ostéoblastique de cellules mésenchymateuses au sein d’un tissu à renouvellement rapide. Cette métaplasie peut être induite par un stimulus soit d’ordre hormonal (coma), soit mécanique par traumatisme répété ou métabolique. Les traumatismes répétés sont probablement des facteurs d’induction de métaplasie cellulaire sur des tissus à croissance rapide. Selon Ekelund [6], les cellules lésées par le traumatisme libèrent des facteurs inducteurs ou des réactions inflammatoires qui induisent une OH. Indépendamment de l’étiologie génétique, l’ossification hétérotopique est censée impliquer trois composantes essentielles: voies inductives de signalisation, les cellules ostéoprogénitrices inductibles et un environnement propices à l’ostéogenèse hétérotopique [8]. Il est difficile de savoir ce qui a précipité la croissance osseuse ectopique chez notre patient de manière retardée. L’acte chirurgical et le traumatisme de haute énergie lui-même seraient une théorie plausible. Cette explication est souvent citée dans les ossifications hétérotopiques du tendon rotulien suite aux alésages de la moelle qui aurait semé le tendon et a provoqué la formation d’os ectopique. Cette théorie a gagné plus d’acceptation lorsque Furlong et al [9], après avoir comparée l’incidence des ossifications hétérotopiques dans les moelles alésées versus non alésées du fémur, ont montré que l’ossification hétérotopique était significativement plus élevée dans le groupe alésé. Ils ont conclu que c’était les débris d’alésage ostéogénique qui ont probablement causé une ossification hétérotopique chez ces patients. Cette théorie a également été soutenue chez les lapins [9]. Cependant, cette théorie a été réfutée par Brumback et al. [10], qui ont montré que l’irrigation abondante du site d’entrée n’affecte pas l’incidence de l’ossification hétérotopique dans l’enclouage fémoral antérograde.

Serait-il possible que les cellules du LLI ossifié possèdent un potentiel d’ostéo-formation comme celles du ligament croisé antérieur et postérieur. En effet, les cellules souches mésenchymateuses ont un grand potentiel pour la régénération tissulaire, ces cellules peuvent être récoltées à partir d’une variété de tissus, il a été précisé que les cellules souches pourraient être isolées à partir des ligaments croisés du genou à la fois antérieur et postérieur (LCA et LCP) de l’homme [11]. Ces cellules sont capables de se différencier en ostéoblastes, chondrocytes et adipocytes sous inductions appropriées. Leurs caractéristiques phénotypiques étaient similaires à celles des cellules de la moelle osseuse [11, 12]. Toutefois, la rupture du LCA et/ou LCP ne s’accompagne pas systématiquement d’ossification. Le mécanisme reste obscure dans notre cas, quel mécanisme ayant déclenché l’ossification isolée de tout le LLI, l’ossification se prolongeait juste en bas de l’insertion du LLI, en dessous du matériel d’ostéosynthèse (Figure 1).

Une autre explication aussi appliquée sur le LCA. L’hémarthrose induite par une blessure du LCA contient des cellules ostéoprogénitrices pouvaient se différencier en ostéoblastes [13]. Cependant, dans l’hémarthrose après lésion isolée du LCA, il n’y a pas de gouttelettes grasses de la moelle osseuse, car il n’y a pas eu d’accès direct pour la moelle osseuse dans l’articulation. Il est possible que ces cellules ostéoprogénitrices puissent être d’origine locale, et peuvent donc être considérées comme des cellules souches [13]. Dans notre cas, serait-il possible que le sang causé par la fracture du plateau tibial contient des gouttelettes grasses dérivées de la moelle osseuse. Par conséquent, de telles gouttelettes contiennent des cellules ostéoprogénitrices. Histologiquement, les cellules mésenchymateuses primitives commencent à se développer dans le tissu ostéoblastique dès 16 heures. A environ 32 heures, la formation de lossification hétérotopique atteint la stimulation maximale. Cette masse augmente de taille entre la 1^ère^ et 2^ème^ semaine [14]. Avec la radiographie standard, l’ossification hétérotopique est identifiable dès la 3^ème^ ou 4^ème^ semaine et peut être suivie tout au long de la maturation [15]. Entre la 5^ème^ et 8^ème^ semaine, le début de l’ostéogenèse peut être identifié, alors qu’entre la 9^ème^ et 12^ème^ semaine, on note la fin de l’ostéogenèse. La scintigraphie osseuse de 0 à 4 semaines, 5 à 8 semaines, et 9 à 12 semaines est corrélée avec les phases positives I à III, phase positive III, et la diminution à la phase III, respectivement [8]. Dans notre cas, l’ossification a été identifiée au 2^ème^ mois en post-opératoire, la maturation osseuse était confirmée par le taux normal des phosphatases alcalines, néanmoins on ne sait pas exactement la date d’apparition.

## Conclusion

Les quatre décennies suivantes n’ont pas connu d’avancée en matière de connaissance de la physiopathologie, la prévention et le traitement. Les ligaments comme les tendons sont susceptibles d’être touchés par l’ossification hétérotopique péri-articulaire. La détermination de facteurs favorisant l’apparition de l’ossification de l’un ou de l’autre mérite des recherches approfondies pour instaurer un traitement adéquat pour enfin préserver le pronostic fonctionnel du genou, voire des autres articulations comme la hanche et le coude. Enfin, la pathogénie de ces ossifications reste inconnue et préoccupe toujours les orthopédistes.
